# A Deep-Learning Algorithm to Predict Short-Term Progression to Geographic Atrophy on Spectral-Domain Optical Coherence Tomography

**DOI:** 10.1001/jamaophthalmol.2023.4659

**Published:** 2023-10-19

**Authors:** Eliot R. Dow, Hyeon Ki Jeong, Ella Arnon Katz, Cynthia A. Toth, Dong Wang, Terry Lee, David Kuo, Michael J. Allingham, Majda Hadziahmetovic, Priyatham S. Mettu, Stefanie Schuman, Lawrence Carin, Pearse A. Keane, Ricardo Henao, Eleonora M. Lad

**Affiliations:** 1 Department of Ophthalmology, Duke University Medical Center, Durham, North Carolina; 2 Department of Biostatistics and Bioinformatics, Duke University Medical Center, Durham, North Carolina; 3 Department of Electrical and Computer Engineering, Duke University, Durham, North Carolina; 4 King Abdullah University of Science and Technology, Thuwal, Saudi Arabia; 5 University College London Institute of Ophthalmology, National Institute for Health and Care Research, Biomedical Research Centre, Moorfields Eye Hospital National Health Services Foundation Trust, London, United Kingdom

## Abstract

**Importance:**

The identification of patients at risk of progressing from intermediate age-related macular degeneration (iAMD) to geographic atrophy (GA) is essential for clinical trials aimed at preventing disease progression. DeepGAze is a fully automated and accurate convolutional neural network–based deep learning algorithm for predicting progression from iAMD to GA within 1 year from spectral-domain optical coherence tomography (SD-OCT) scans.

**Objective:**

To develop a deep-learning algorithm based on volumetric SD-OCT scans to predict the progression from iAMD to GA during the year following the scan.

**Design, Setting, and Participants:**

This retrospective cohort study included participants with iAMD at baseline and who either progressed or did not progress to GA within the subsequent 13 months. Participants were included from centers in 4 US states. Data set 1 included patients from the Age-Related Eye Disease Study 2 AREDS2 (Ancillary Spectral-Domain Optical Coherence Tomography) A2A study (July 2008 to August 2015). Data sets 2 and 3 included patients with imaging taken in routine clinical care at a tertiary referral center and associated satellites between January 2013 and January 2023. The stored imaging data were retrieved for the purpose of this study from July 1, 2022, to February 1, 2023. Data were analyzed from May 2021 to July 2023.

**Exposure:**

A position-aware convolutional neural network with proactive pseudointervention was trained and cross-validated on Bioptigen SD-OCT volumes (data set 1) and validated on 2 external data sets comprising Heidelberg Spectralis SD-OCT scans (data sets 2 and 3).

**Main Outcomes and Measures:**

Prediction of progression to GA within 13 months was evaluated with area under the receiver-operator characteristic curves (AUROC) as well as area under the precision-recall curve (AUPRC), sensitivity, specificity, positive predictive value, negative predictive value, and accuracy.

**Results:**

The study included a total of 417 patients: 316 in data set 1 (mean [SD] age, 74 [8]; 185 [59%] female), 53 in data set 2, (mean [SD] age, 83 [8]; 32 [60%] female), and 48 in data set 3 (mean [SD] age, 81 [8]; 32 [67%] female). The AUROC for prediction of progression from iAMD to GA within 1 year was 0.94 (95% CI, 0.92-0.95; AUPRC, 0.90 [95% CI, 0.85-0.95]; sensitivity, 0.88 [95% CI, 0.84-0.92]; specificity, 0.90 [95% CI, 0.87-0.92]) for data set 1. The addition of expert-annotated SD-OCT features to the model resulted in no improvement compared to the fully autonomous model (AUROC, 0.95; 95% CI, 0.92-0.95; *P* = .19). On an independent validation data set (data set 2), the model predicted progression to GA with an AUROC of 0.94 (95% CI, 0.91-0.96; AUPRC, 0.92 [0.89-0.94]; sensitivity, 0.91 [95% CI, 0.74-0.98]; specificity, 0.80 [95% CI, 0.63-0.91]). At a high-specificity operating point, simulated clinical trial recruitment was enriched for patients progressing to GA within 1 year by 8.3- to 20.7-fold (data sets 2 and 3).

**Conclusions and Relevance:**

The fully automated, position-aware deep-learning algorithm assessed in this study successfully predicted progression from iAMD to GA over a clinically meaningful time frame. The ability to predict imminent GA progression could facilitate clinical trials aimed at preventing the condition and could guide clinical decision-making regarding screening frequency or treatment initiation.

## Key Points

**Question:**

Can a convolutional neural network-based deep learning algorithm predict progression from intermediate age-related macular degeneration (iAMD) to geographic atrophy (GA) from a volumetric spectral-domain optical coherence tomography (SD-OCT) scan?

**Findings:**

In this cohort study of 417 patients, a convolutional neural network accurately predicted eyes that progressed from iAMD to GA within 1 year using volumetric SD-OCT scans. Simulations using the convolutional neural network for clinical trial recruitment of patients at risk for disease progression resulted in a greater yield in identifying patients progressing to GA in the trial cohort.

**Meaning:**

The findings in this study suggest that automated prediction of imminent GA progression could facilitate clinical trials aimed at preventing disease and guide clinical decision-making regarding screening frequency or treatment initiation.

## Teaser

This cohort study describes a deep-learning algorithm based on spectral-domain optical coherence tomography to predict 1-year progression from intermediate age-related macular degeneration to geographic atrophy.

## Introduction

Geographic atrophy (GA) is the advanced nonexudative form of age-related macular degeneration (AMD) characterized by loss of photoreceptors, retinal pigment epithelium, and choriocapillaris.^1,2^ These changes often begin in the perifovea and later progress to the fovea with debilitating, irreversible outcomes for central vision.^3^ GA is preceded by the intermediate stage of dry AMD (iAMD), which usually has minimal effect on visual acuity. GA affects more than 5 million individuals worldwide, a figure that is steadily rising as the median age worldwide increases.^4,5^

However, an impediment to the study of preventative treatment is the low likelihood of progressing from iAMD to GA: the per-year incidence of progression is estimated to be 0.75% to 3.67%.^6,7,8^ Consequently, without a means to identify the patients most likely to progress, clinical studies of GA prevention are hindered by the need for long study periods and large patient cohorts. Additionally, in a potential future with therapeutics that prevent progression from iAMD to GA, predicting near-term progression to GA would be valuable in targeting treatment to the patients who stand to benefit. Finally, more frequently examining iAMD patients at high-risk of progression to GA would allow the early initiation of currently available therapeutics for greater preservation of retinal tissue. Thus, we sought to use deep learning to predict the progression from iAMD to GA. We aimed to create an algorithm that did not require human annotation or expert feature selection; generalized to multiple spectral-domain optical coherence tomography (SD-OCT) devices, including current standard-of-care models; was validated on data obtained during routine patient care; made predictions on a clinically meaningful timeframe; and was automated end-to-end allowing for the screening of large patient databases without the need for human intervention.

## Methods

### Ethics and Institutional Governance Approvals

This study was reviewed and approved by the Duke University institutional review board. Patient consent for inclusion of data was waived for this retrospective analysis, which did not alter standard patient care procedures. Patient data were deidentified and precautions were taken as per Duke University institutional review board protocol to ensure the security of protected health information and other study data. The protocol followed tenets of human research as presented in the Declaration of Helsinki. The study followed the Strengthening the Reporting of Observational Studies in Epidemiology (STROBE) reporting guideline.

### Data Sets and Clinical Taxonomy

The study involved 3 independent data sets (Table). Data set 1 was collected in the course of the Age-Related Eye Disease Study 2 (AREDS2) Ancillary Spectral-Domain Optical Coherence Tomography (A2A) study,^6^ an ancillary observational prospective study of a subset of eyes from the multisite AREDS2 study.^9,10^ Bioptigen (Research Triangle Park, North Carolina) SD-OCT volumes (6.7 mm × 6.7 mm, 100 B-scans per volume) were obtained at 4 participating institutions: Emory University Eye Center, Atlanta, Georgia; Devers Eye Center, Portland, Oregon; Duke Eye Center, Durham, North Carolina; and the National Eye Institute, Bethesda, Maryland.^3,4^ Details of the study have been previously published.^9^ The resultant data set for model development and cross-validation from the A2A study (data set 1) consisted of 304 volumetric SD-OCT scans of eyes with GA in the present scan; 60 SD-OCT volumes of eyes with iAMD that progressed to GA at an encounter 1 year later (progression); and 721 SD-OCT volumes of eyes with iAMD that did not progress to GA at an encounter 1 year later (nonprogression). For all data sets, OCT-GA was defined as the presence of the following 3 criteria: (1) retinal pigment epithelium atrophy or absence, (2) choroid enhancement, and (3) outer plexiform layer dipping toward the retinal pigment epithelium over an area of at least 175 μm as defined in the AREDS2 A2A study and cited in prior publications.^9,11,12^ This definition preceded the establishment of the Classification of Atrophy Meeting, Complete Retinal Pigment Epithelial and Outer Retinal Atrophy (cRORA) as a clinical and research definition of GA on OCT. Nevertheless, there were no cases of iAMD that also met criteria for cRORA, and all but 1 case that progressed to OCT-GA also met the definition of progression to cRORA at the same time point. For all data sets, iAMD was defined as extensive medium drusen (63-125 μm) or large drusen (≥126 μm) with no evidence of advanced exudative or nonexudative AMD per previous definitions, and all eyes with exudative AMD were excluded from the study.^13,14,15^

**Table.** Characteristics of Data Sets 1, 2, and 3

Characteristic	Data set 1	Data set 2	Data set 3
Role in model development	Training, cross validation	External validation	External validation
Setting of data collection	Clinical study	Routine patient care	Routine patient care
Location of acquisition	Atlanta, Georgia; Portland, Oregon; Bethesda, Maryland; Durham, North Carolina	Durham, South Durham, Raleigh, North Carolina	Morrisville, Cary, North Carolina
SD-OCT device	Bioptigen SD-OCT	Heidelberg spectralis	Heidelberg spectralis
No. of patients	316	53	48
No. of eyes	316	53	48
No. of OCT volumes	1085	53	48
iAMD to iAMD, No. (%)	721 (66.5)	30 (56.6)	22 (45.8)
iAMD to GA, No. (%)	60 (5.5)	23 (43.4)	26 (54.2)
GA to GA, No. (%)	304 (28.0)	0	0
Data labeling	Grading in certified reading center	Consensus grading by 3 ophthalmologists	Consensus grading by 3 ophthalmologists
Demographic characteristics			
Age, mean (SD), y	74 (8)	83 (8)	81 (8)
Sex, No. (%)			
Female	185 (59)	32 (60)	32 (67)
Male	131 (41)	16 (33)	21 (40)
Race and ethnicity, No. (%)^a^			
Asian	3 (1)	1 (2)	0
Black	5 (2)	1 (2)	2 (4)
Hispanic/Latino	NA	0	0
Non-Hispanic/Latino	NA	50 (94)	47 (98)
White	298 (95)	50 (94)	46 (96)
Other/unknown^a^	10 (3)	1 (2)	0
Declined to answer	NA	3 (6)	1 (2)

Abbreviations: GA, geographic atrophy; iAMD, intermediate age-related macular degeneration; NA, not applicable; OCT, optical coherence tomography; SD-OCT, spectral-domain optical coherence tomography.

^a^ Race and ethnicity data were acquired via patient self-reported data as reflected in the electronic medical record. As such, the category of other reflects the record and cannot be further defined herein.

Validation SD-OCT scans were obtained from routine outpatient encounters within the Duke University Health System from July 2008 to August 2015 (Table). The first independent validation data set (data set 2) was composed of Spectralis (Heidelberg, Germany) SD-OCT scans (8.7 mm × 7.2 mm, 61 B-scans per volume) obtained from the Main Duke Eye Center (Durham, North Carolina) and 2 regional satellite practices (Raleigh, North Carolina, and South Durham, North Carolina). Data sets 2 and 3 were collected between July 1, 2022, and February 1, 2023. Under an adjudicated consensus labeling system, 3 experienced ophthalmologists (E.D., E.K., E.L.) evaluated volumetric SD-OCT scans, near-infrared reflectance imaging, and, where available, fundus autofluorescence images and other multimodal imaging for cases of iAMD or GA. For eyes with GA, preceding SD-OCT scans were obtained and labeled as to the number of days to the first SD-OCT scan depicting GA designated as the date of progression. For eyes without GA, SD-OCT scans were labeled as to the number of days in the future that a subsequent SD-OCT ascertained that the eye had not developed GA. For patients in whom both eyes had iAMD or GA, 1 eye was randomly selected for inclusion. Data set 2 included 53 patients: 23 eyes with iAMD that progressed to GA within 13 months and 30 with iAMD that did not progress to GA over the same period. The prediction interval was extended from 12 months to 13 since routine monitoring of iAMD often recurred at just greater than a 12-month interval. A second independent validation data set (data set 3) was collected from 2 additional regional satellite practices within the Duke University Health System located in different cities (Morrisville, North Carolina, and Cary, North Carolina) and with distinct medical staff and SD-OCT devices from the clinics from which data set 2 were obtained. The methods applied to data set 2 were also applied to data set 3. Data set 3 included 48 patients: 26 eyes with iAMD that progressed to GA and 22 eyes that did not progress to GA during the same time period (Table). Since there were no a priori estimates of the expected effect size or performance of the model, an initial arbitrary but logistically feasible target of 50 eyes was set for each data set, balanced between those that progressed to iAMD and those that did not. Data were analyzed from May 2021 to July 2023.

### Model

A multiview convolutional neural network architecture^16^ based on and initialized with parameters from the Inception version3 neural network^17^ was pretrained on natural images from ImageNet.^18^ Additional domain-specific pretraining was performed with a publicly available SD-OCT data set with multi-task learning.^18,19^ The position-aware model used a transformation layer to embed the position identifier into a 6-dimensional positional feature vector *e_i_*. Then, the feature vector *f_i_* and positional feature *e_i_* were concatenated and fed into a fully connected layer to obtain *a_i_* = *FC*_2_([*f_i_, e_i_*]), which were progressed to attention weights *w_i_* by feeding the *a_i_* into a softmax function, so:
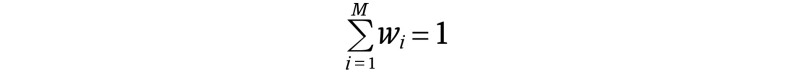
The final probability of GA for a given SD-OCT volume was the weighted summation of the attention weights *w_i_* and corresponding preclassification probabilities *p_i_* for all scans 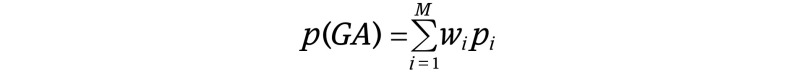
(eFigure 1 in Supplement 1). The model was trained in a contrastive learning manner with proactive pseudointervention learning as previously described (eFigure 1 in Supplement 1).^20^

The model was coded in PyTorch and trained with the Adam Optimizer^21^ on a GPU TITAN Xp for 100 epochs with a learning rate of 0.0005 for pretrained feature exactor (0.005 for fully connected layers) and a decay of 0.5 applied to the learning rate at every 10 epochs. Model development was performed on 108 500 SD-OCT B-scans of 512 × 1000 pixels corresponding to 1085 individuals, 28% of which (30 400 scans) correspond to patients with GA (data set 1) (Table). Model performance was estimated via 5-fold cross-validation with standard deviation. The statistical significance of the difference between receiver-operating characteristic (ROC) curves for different models was quantified with the DeLong test.^22^ Confusion matrices and their summaries (sensitivity, specificity, positive predictive value, negative predictive value, and accuracy) obtained by thresholding the prediction values from the model with values estimated by Youden index.^23^ To visually interpret model predictions, the model generated attention maps via weight backpropagation,^20^ which probabilistically masks out regions of the scan that do not contribute to the ability of the model to predict GA.

In order to justify the design choices in the proposed multi-scan position-aware model trained with proactive pseudointervention, we performed ablation studies to assess the contribution of each component (eTable 1 in Supplement 1). Additionally, human-annotated features were fed into the preprediction layer of the model to determine if it could improve model accuracy (eTable 2 in Supplement 1). These human-annotated features have been previously published.^6,10,11,24,25,26^ For model validation, SD-OCT volume scans from the independent validation data sets 2 and 3 were input to the final model (multiscan, position-aware model trained with proactive pseudointervention) after contrast limited adaptive histogram equalization image normalization. Additional details may be found in eMethods in Supplement 1.

## Results

**Figure 1.**
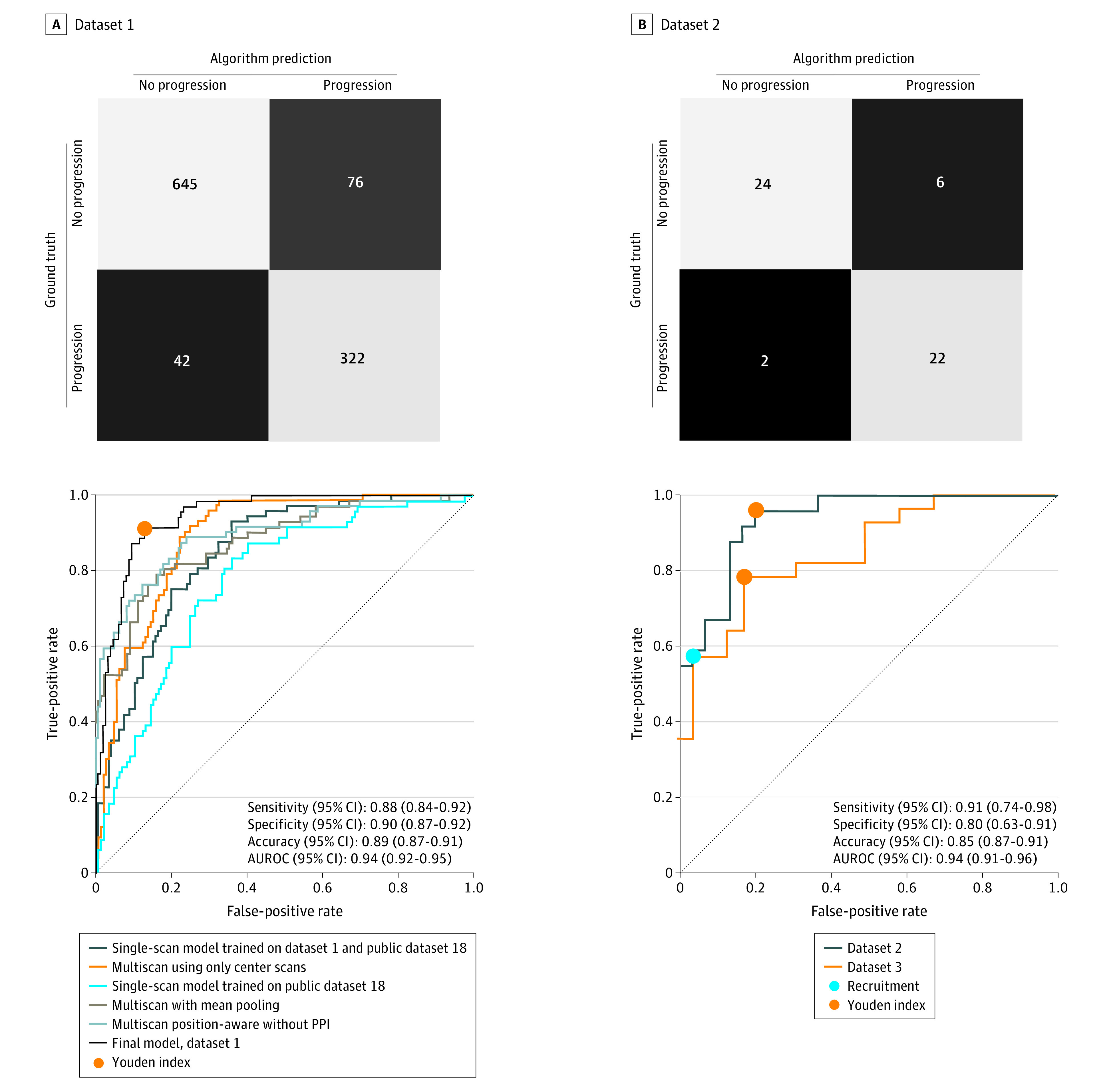
Prediction of Progression From Intermediate Age-Related Macular Degeneration to Geographic Atrophy Within 1 Year Across 3 Independent Data Sets Rows of the confusion matrices represent ground truth labels and columns represent model predictions. Assignments were obtained by thresholding-predicted geographic atrophy probabilities with thresholds chosen by Youden index. Area under the receiver operating characteristic curves (AUROCs) for data set 1 depict the final model in addition to lines for ablation design-based studies of the model. AUROCs for data sets 2 and 3 display a high-specificity operating point selected for clinical trial recruitment in addition to 1 identified by Youden index.

The study included a total of 417 patients: 316 in data set 1 (mean [SD] age, 74 [8]; 185 [59%] female), 53 in data set 2, (mean [SD] age, 83 [8]; 32 [60%] female), and 48 in data set 3 (mean [SD] age, 81 [8]; 32 (67%] female). The convolutional neural network-based deep-learning model used in this study for prediction of progression from iAMD to GA was trained and cross-validated on SD-OCT volumes from data set 1 (Table; eTable 1 in Supplement 1). The prediction of progression from iAMD to GA within 1 year yielded an area under the ROC curve (AUROC) of 0.94 (95% CI, 0.92-0.95) and area under the precision-recall curve (AUPRC) of 0.90 (95% CI, 0.85-0.95) on 5-fold cross validation. An optimal threshold value was obtained by Youden index^23^ resulting in the following performance values for the prediction of GA 1 year later: sensitivity 0.88 (95% CI, 0.84-0.92), specificity 0.90 (95% CI, 0.87-0.92), positive predictive value 0.82 (95% CI, 0.75-0.89), negative predictive value 0.94 (95% CI, 0.89-0.97), and accuracy of 0.89 (95% CI, 0.87-0.91) (Figure 1). For identification of GA in the eye at the current time of acquisition, we obtained AUROC of 0.95 (95% CI, 0.89-0.98), AUPRC of 0.91 (95% CI, 0.87-0.95), sensitivity of 0.93 (95% CI, 0.87-0.99), specificity of 0.85 (95% CI, 0.78-0.92), positive predictive value of 0.71 (95% CI, 0.65-0.76), negative predictive value of 0.97 (95% CI, 0.95-0.99), and accuracy of 0.88 (95% CI, 0.83-0.94).

To assess the performance of the fully automated model relative to one supplemented with human-selected image features, we compared a version of the final model trained with human-annotated SD-OCT features associated with progression to GA to a version without the human-annotated features (eTable 2 in Supplement 1).^6^ The model with the additional human-annotated features (eTable 1 in Supplement 1) produced an AUROC of 0.95 (95% CI, 0.92-0.95) for the 1-year prediction of GA, exceeding the fully automated model by a margin of 0.01 AUROC (95% CI, 0.02-0.03; *P* = .19).

The model was further validated on an independent data set (data set 2) (Table) obtained during routine patient care on a separate SD-OCT device (Heidelberg Spectralis). Data set 2 consisted of 53 SD-OCT volumes from eyes with iAMD, 23 of which progressed to GA within the subsequent 13 months. AUROC on the validation set was 0.94 (95% CI, 0.91-0.96) for prediction of GA at 13 months (AUPRC, 0.92 [95% CI, 0.89-0.94]; sensitivity, 0.91 [95% CI, 0.74-0.98]; specificity, 0.80 [95% CI, 0.63-0.91]; positive predictive value, 0.78 [95% CI, 0.70-0.85]; negative predictive value, 0.92 [95% CI, 0.90-0.95]; and accuracy, 0.85 [95% CI, 0.87-0.91]) (Figure 1). The external validation data set did not exclude cases on the basis of macular comorbidities, and as a result, 13 cases (24.2%) also had cystoid macular edema, peripapillary atrophy, vitreomacular traction, or epiretinal membrane with pucker. For identification of GA at the current time in data set 2, we obtained an AUROC of 0.97 (95% CI, 0.96-0.99).

**Figure 2.**
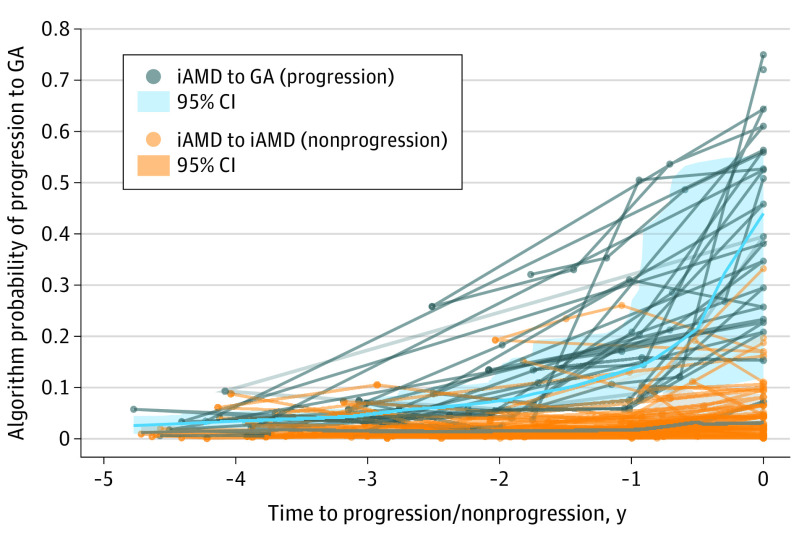
Temporal Specificity of the Model Shown by Prediction of Likelihood of Progression From Intermediate Age-Related Macular Degeneration (iAMD) to Geographic Atrophy (GA) Across 5 Years From the Date of GA Progression or Nonprogression The x-axis represents years prior to progression or censoring for those that did not progress. Lightweight lines connect spectral-domain optical coherence tomography scans from the same patient across time, whereas the bold lines average probabilities from all cases within the 2 groups.

Looking beyond 1-year predictions, when the model was given SD-OCT scans obtained from eyes up to 2 years prior to GA progression, the AUROC remained high at 0.88 (95% CI, 0.80-0.96). However, the ability of the model to predict GA diminished rapidly beyond 24 months, and the model could not distinguish SD-OCT scans from eyes that would progress in 3 to 5 years from those that would not progress in 3 to 5 years or more (Figure 2).

**Figure 3.**
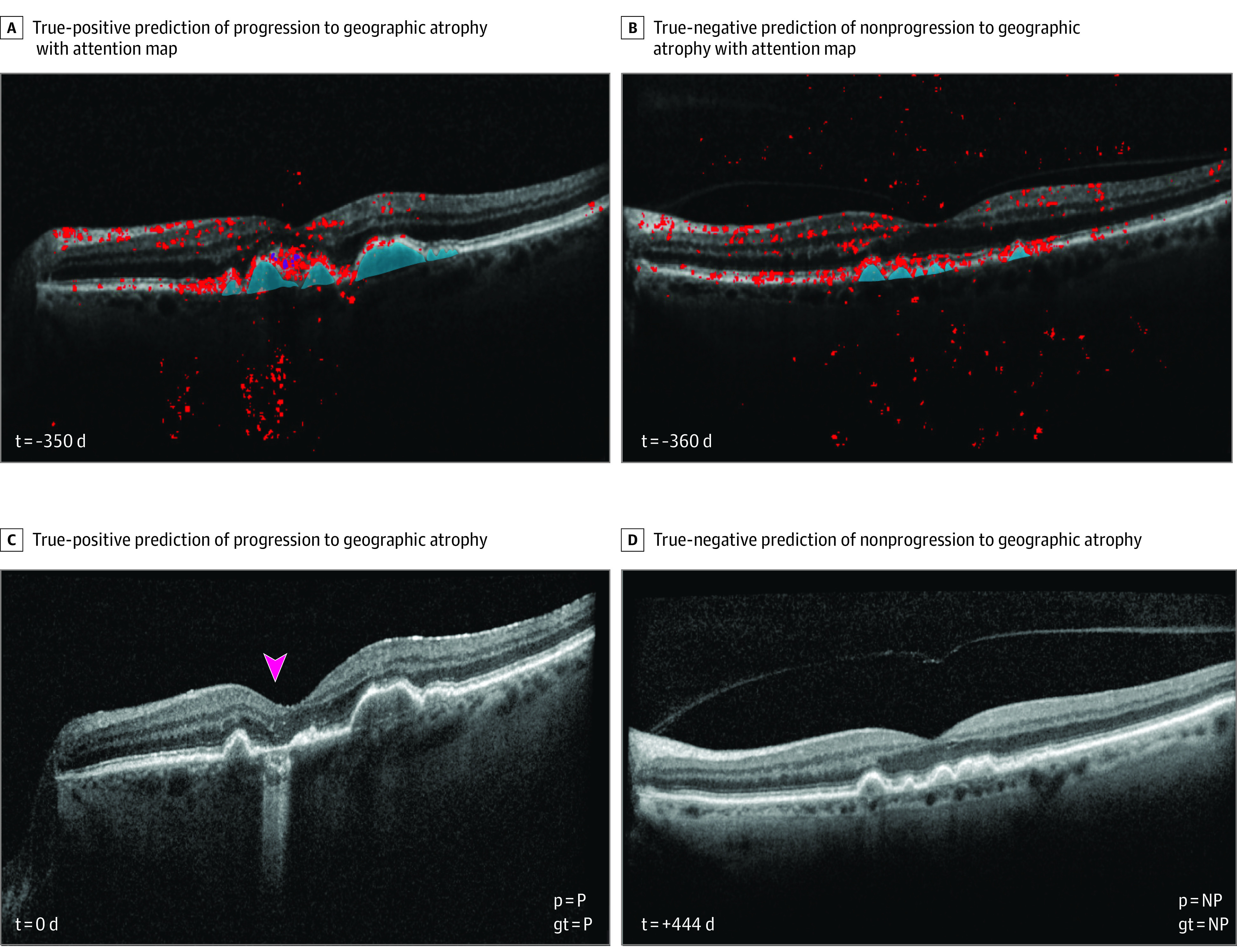
Example Predictions From a Deep-Learning Algorithm A and C, True positive prediction of progression to geographic atrophy (GA). The attention map shows clustering around the pigment epithelial defect with overlying hyperreflective foci that collapses into a GA lesion (arrowhead) less than 1 year later. B and D, True negative prediction of nonprogression to GA shows model attention directed to several pigment epithelial defects as well as diffusely distributed along the outer retina. At 26 months after prediction, the eye remained without GA.

We sought to further understand the performance of the model by using weight backpropagation to generate attention maps for SD-OCT volumes (Figure 3; eFigures 2-5 in Supplement 1). In these attention maps, red dots highlight areas that the model identifies as most salient for the prediction of GA or progression to GA. In eyes with current GA (eFigure 2A in Supplement 1), the red dots were concentrated in the GA lesion area associated with the atrophic outer retina, the Bruch membrane, underlying choriocapillaris and choroid, and often in the neurosensory retina over the GA lesion in a vertical distribution through the nerve fiber layer. In eyes with iAMD that would progress to GA in 1 year (Figure 3; eFigures 2-5 in Supplement 1), the red dots were predominantly found in large pigment epithelial detachments, especially those with overlying hyperreflective foci and early choroidal hypertransmission. This reflected the fact that in 303 cases (83.3%) of progression in our data set, the initial GA lesion arose following the collapse of a pigment epithelial detachment; in many of the remaining 61 cases (16.7%), there were not scans sufficiently far into the past to establish that the atrophic lesion had not arisen from a previously collapsed pigment epithelial detachment. Among nonprogressing eyes, the weight backpropagation maps showed attention distributed more diffusely across larger areas of retinal pigment epithelium and drusen rather than clustered around suspicious lesions (Figure 3). Further supporting the face validity of the model, the extrafoveal SD-OCT B-scans where GA lesions rarely appeared showed diffusely scattered attention mapping and low predictive value (eFigures 4 and 6 in Supplement 1). Prediction errors by the model showed a less coherent pattern of attention mapping. In 1 example of a false-positive result, attention was more diffusely distributed similar to the pattern of attention seen in true negative cases, and in fact, progression to GA occurred just 1 year later than the prediction (eFigure 5 in Supplement 1). In an example case of a false negative, attention was clustered on unrelated image features rather than a pigment epithelial detachment that would collapse into a GA lesion 12 months later (eFigure 5 in Supplement 1).

**Figure 4.**
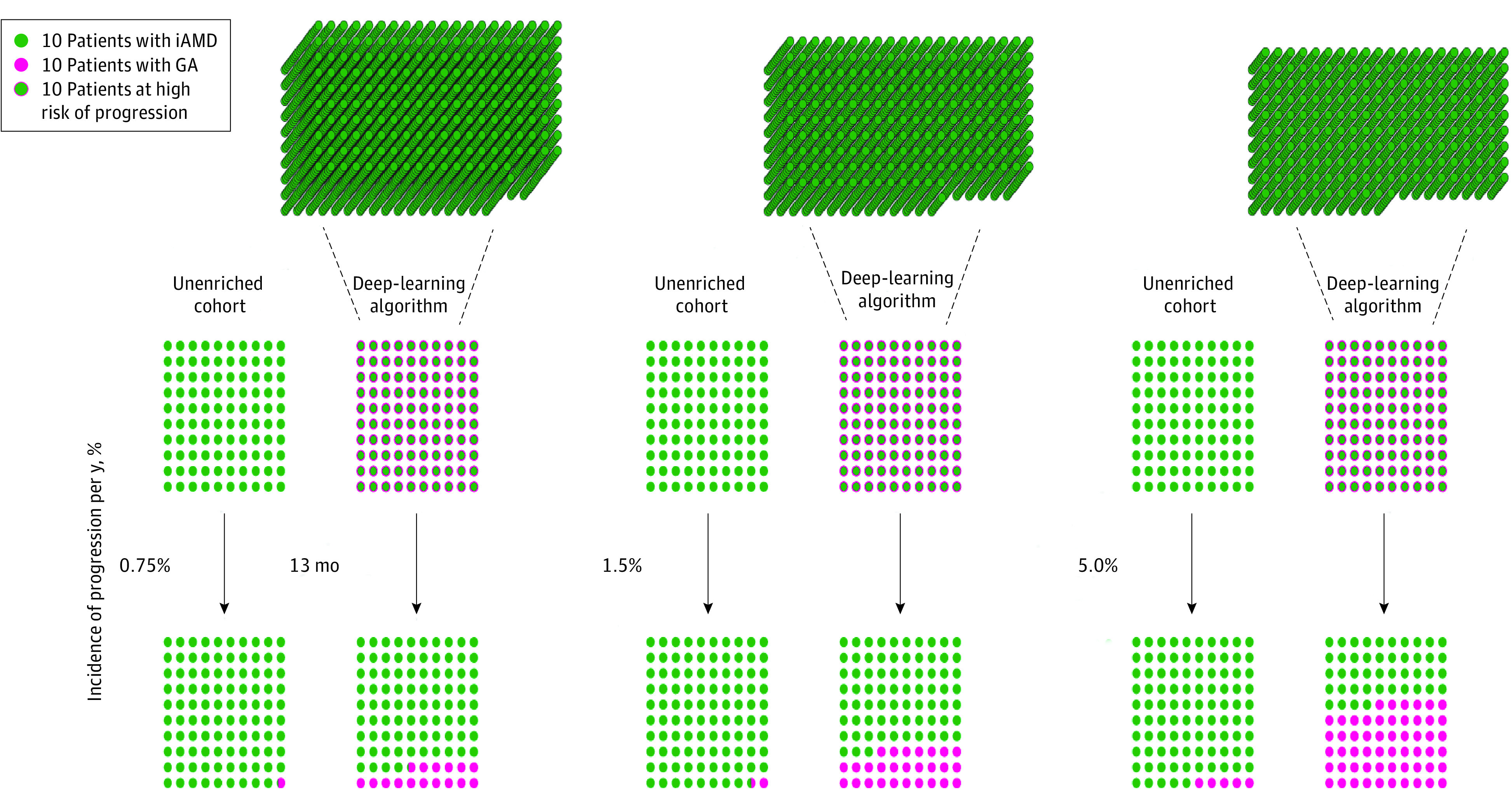
Clinical Trial Enrichment of Patients Progressing From Intermediate Age-Related Macular Degeneration (iAMD) to Geographic Atrophy (GA) Using a Deep-Learning Algorithm A cohort of patients recruited from the unenriched population of patients with iAMD was compared to a cohort screened and selected from large clinical databases. Comparisons are shown at 3 different estimates of incidence of progression from iAMD to GA per year (0.75%, 1.5%, and 5.0%).

In data set 2, we selected a high-specificity operating point optimized for high throughput autonomous patient screening for clinical-trial recruitment. At this operating point, specificity was 0.98 (95% CI, 0.94-1.00) and sensitivity was 0.59 (95% CI, 0.53-0.63) (Figure 1). We calculated the enrichment that could be achieved in patients progressing from iAMD to GA if the model were used to screen and enroll 1000 patients for a hypothetical 1-year clinical trial. Depending on the baseline incidence of iAMD to GA progression in the population, use of the model would lead to an 11.2- to 20.7-fold enrichment in progressing patients in this data set (Figure 4; eTable 3 in Supplement 1). Since the model would need to be autonomously applied to multiple image databases in the course of clinical-trial recruitment, we tested its performance in data set 3 at the same operating threshold. At this value, the high-specificity operating point showed a specificity of 0.96 (95% CI, 0.95-0.99), sensitivity of 0.60 (95% CI, 0.49-0.68), and an 8.3- to 12.2-fold enrichment depending on baseline prevalence of progression (eTable 3 in Supplement 1).

## Discussion

Progression from iAMD to GA represents a transition from a largely asymptomatic condition to one that may devastate central vision. However, since fewer than 1 in 21 will progress from iAMD to GA each year, identifying at-risk individuals is a crucial but difficult task.^6,7,8^ In this cohort study, we describe a convolutional neural network-based deep-learning algorithm designed to predict the progression from iAMD to GA from SD-OCT volumes.

Although a number of previous research efforts have applied machine learning to predicting progression to GA, to our knowledge, there has not been an algorithm with high accuracy, single-modality input, full automation, and external validation.^6,27,28,29,30^ Recent publications have also used deep learning to automatically detect pre-GA lesions, like incomplete retinal pigment epithelium and outer retinal atrophy or nascent GA, from SD-OCT images. However, the predictive utility and reproducibility of these markers for progression to GA remains in contention.^31,32,33,34^ For instance, Wu et al^35^ found that incomplete retinal pigment epithelium and outer retinal atrophy did not contribute any greater predictive power beyond that offered by nascent geographic atrophy in predicting GA progression. Indeed, only 3% of observed eyes with incomplete retinal pigment epithelium and outer retinal atrophy progressed to GA within 36 months in the study, compared to 93.1% of eyes with incomplete retinal pigment epithelium and outer retinal atrophy in the initial study.^32,34^ These discrepant results may be due in part to the challenging of achieving interreader agreement for incomplete retinal pigment epithelium and outer retinal atrophy even among experts. Research is underway to address this critical unmet need to predict progression from iAMD to GA.^36^

Our algorithm exceeds previous efforts in several respects. First, the model was trained on the high-quality AREDS2-A2A data collected under clinical trial protocols and is, to our knowledge, the largest data set of data depicting progression from iAMD to GA. Second, our model was validated on 2 independent data sets collected in the course of routine patient care. Third, the model’s face validity was confirmed with weight backpropagation attention maps that highlighted the pathological regions of AMD as the most salient for model prediction. Fourth, the model required input of a single SD-OCT volume for prediction, an imaging modality available for every patient encounter in a retina health care provider’s office. Fifth, the model generalized across SD-OCT devices, including Heidelberg Spectralis, a standard-of-care device for both patient care and clinical trials. The definition of GA was recently updated for SD-OCT criteria instead of color fundus photography, and it is likely that patient care and clinical trials in the future will continue to be carried out primarily with this imaging modality.^37,38,39^ Sixth, the model demonstrated excellent performance on the external data sets. Seventh, the model showed temporal specificity for short-term conversion within 1 to 2 years. Seventh, we used an end-to-end approach, in that the model receives an SD-OCT volume as input and produces binary predictions without the need for human selection of image features, input of clinical and demographic data, or other manual steps. Adding data features generated by a clinical-trial reading center did not improve model performance.

This latter strength of the algorithm is essential for its application to clinical trials or patient care. Investigators seeking to test new therapies to prevent the progression from iAMD to GA could apply the model to large databases of SD-OCT volumes and return a list of patients likely to undergo progression during the 1- to 2-year duration of the trial. Depending on rates of disease progression within a population, the use of our algorithm at its high-specificity operating point could lead to an 8.3- to 12.2-fold increase in the yield of patients whose eyes are progressing when applied to external databases of images collected during routine patient care (Figure 4; eTable 3 in Supplement 1). As 1 example, at a commonly accepted incidence of progression from iAMD to GA of 3.0% per year, a clinical trial that enrolled 1000 patients with iAMD for a 1-year trial could expect just 30 of them to progress to GA, whereas using a deep-learning algorithm to screen patients for trial enrollment would yield 292 progressing patients per year—a nearly 10-fold increase. This order of magnitude or greater enrichment would facilitate clinical studies that today may be infeasible.

### Limitations

Our study has several limitations. First, although the data involved in the study represent, to our knowledge, the largest published cohort of patients progressing to GA, it is nevertheless a small number of cases for deep learning (by comparison, ImageNet, a natural image data set widely used to develop deep learning algorithms, contains over 14 million images). The size of the training data also limits the nature of the prediction of GA progression. A nonbinary output from the model, for instance, a prediction of months or years to GA progression, would be superior to binary class prediction. However, such a model would require a larger training set to achieve a high level of accuracy. A larger training set may also allow for additional learning that could extend the prediction beyond 2 years for greater inclusion of pathology.

Another limitation is that the validation data set was manually assembled by a human reader from billing and diagnosis codes. Large image databases may contain a wider variety of ocular phenotypes than what was encountered by a deep-learning algorithm in the current study that may affect performance. Nevertheless, the algorithm had excellent performance in the presence of ocular comorbidities for both true positive and true negative cases.

As another challenge to the prediction of GA, the definition of the disease continues to evolve, particularly as newer imaging modalities like SD-OCT supplant older modalities like color fundus photography. A more recent GA-like entity defined by cRORA has been used as an end point in several studies.^39,40^ We believe that our algorithm would be sufficient to identify the progression from iAMD to cRORA. In fact, 97.3% of cases of progression to GA in our external validation data sets also met the definition of cRORA; in only one case, a new GA lesion measured 200 μm, which fulfilled the definition of GA by OCT but not cRORA. Moreover, we plan to expand the capacity of our algorithm to encompass additional clinical trial end points.

Additionally, the development data set was class imbalanced. However, the AUPRC, a sensitive measure of performance in imbalanced data sets, was high for the internal data set, and moreover, the 2 external validation data sets were balanced between classes. Additional training and validation of the model will be sought through external collaborations for the improvement of prediction accuracy and confirmation of the algorithm’s generalization to real-world databases. This is especially important since the racial and ethnic diversity of the training data was low, although this challenge extends beyond our data sets due to the demographic predilections of AMD. Nevertheless, this work provides an important foundation for future larger scale efforts, most desirably a prospective validation study.

## Conclusions

The findings in this study present the development of a novel, fully automated deep-learning algorithm to detect the presence of GA secondary to AMD in SD-OCT volumetric scans and to predict progression from iAMD to GA within 1 year. The multiscan position-aware model trained with proactive pseudointervention had excellent performance characteristics that were equivalent to a similar algorithm that was also trained on expert-defined features without requiring the costly and labor-intensive process of image annotation. The value of a deep-learning algorithm to predict progression to GA is 3-fold. First, it may facilitate enrollment for clinical trials for iAMD through high-throughput screening of large databases to identify patients with iAMD at high risk of imminent progression to GA. Second, if an effective treatment to prevent progression to GA becomes available in the future, this algorithm may help physicians decide which patients with GA would derive the greatest benefit from treatment. Third, in the current setting of an approved therapy to slow GA, the algorithm can identify patients who should be monitored more frequently so that therapy can be initiated at the onset of disease.

## Notes

Supplement 1. eTable 1 eTable 2 eTable 3 eFigure 1 eFigure 2 eFigure 3 eFigure 4 eFigure 5 eFigure 6 eMethods eReferences

Supplement 2. Data sharing statement
