# Supplementary material for: A Deep-Learning Algorithm to Predict Short-Term Progression to Geographic Atrophy on Spectral-Domain Optical Coherence Tomography
Source: JAMA Ophthalmol. 2023 Oct 19;141(11):1052–61. doi: 10.1001/jamaophthalmol.2023.4659 (PMC10587827; doi:10.1001/jamaophthalmol.2023.4659)
Supplement: Supplement 1. — eTable 1 eTable 2 eTable 3 eFigure 1 eFigure 2 eFigure 3 eFigure 4 eFigure 5 eFigure 6 eMethods eReferences [file jamaophthalmol-e234659-s001.pdf]

Supplemental Online Content

Dow ER, Jeong HK, Arnon Katz E, et al. A deep-learning algorithm to predict short-term progression to geographic atrophy on spectral-domain optical coherence tomography. *JAMA Ophthalmol*. Published online October 19, 2023. doi:10.1001/jamaophthalmol.2023.4659

eTable 1

eTable 2

eTable 3

eFigure 1

eFigure 2

eFigure 3

eFigure 4

eFigure 5

eFigure 6

eMethods

eReferences

This supplemental material has been provided by the authors to give readers additional information about their work.

© 2023 Dow ER et al. *JAMA Ophthalmology.*

eTable 1. Cross-validated performance characteristics of the final proposed model (in bold) for prediction of geographic atrophy (GA) progression within the next year. The difference in the area under the receiver operating characteristics curve (AUROC) between the proposed model and the next closest autonomous model was statistically significant ($p < .001$) using the Delong test for receiving operating characteristic comparisons.[22] The difference between the proposed model and the same model supplemented with human-annotated features was not statistically significant ($p = .19$).

Model	AUC (SD)
Fully Autonomous:	
**Multi-scan position-aware model with PPI**	0.937 (0.017)
Multi-scan model using only center scans	0.890 (0.047)
Multi-scan position-aware model without PPI	0.877 (0.040)
Multi-scan model with mean pooling	0.862 (0.030)
Single-scan model trained on Data Set 1 and (Kermany 2018)	0.840 (0.030)
Single-scan model trained on public SD-OCT data set (Kermany 2018)	0.781 (0.026)
Single-scan model trained with natural images (Deng 2009)	0.650 (0.024)
Human Annotation:	
Multi-scan position-aware model trained with PPI and human-annotated features	0.945 (0.018)

© 2023 Dow ER et al. *JAMA Ophthalmology.*

eTable 2. A description of manually annotated SD-OCT features from Data Set 1. Features were used to provide further training of DeepGAze in addition to the strategy without annotated data as described previously. The version of DeepGAze trained with manually annotated features did not show improvement compared to the version without the features. All other experiments were performed with the version of DeepGAze trained without manually annotated features.

**Description of Human-Annotated Features from SD-OCT**
OCT Volume of Retinal Pigment Epithelium Drusen Complex
OCT Volume of Neurosensory Retinal Thickness
OCT Volume of Drusen
OCT Volume of Retinal Pigment Epithelium Drusen Complex Abnormal Thinning
OCT Presence of Vitreomacular Attachment
OCT Presence of Epiretinal Membrane
OCT Presence of Vitreomacular Attachment or Epiretinal Membrane
OCT Presence of Cystoid Macular Edema
OCT Presence of Cystoid Macular Edema and Geographic Atrophy
OCT Presence of Subretinal Fluid
OCT Presence of Subretinal Fluid: Turbid/Reflective Subretinal Material
OCT Presence of Subretinal Fluid: Reflective Changes in Retinal Above Subretinal Fluid
OCT Presence of Other Subretinal Lesion
OCT Presence of Retinal Pigment Epithelium Elevation (Drusen/PED): Retinal Pigment Epithelium Elevation with Low Refectivity: Less Than or Equal to Photoreceptor Layer
OCT Presence of Retinal Pigment Epithelium Elevation (Drusen/PED): Retinal Pigment Epithelium Elevation with High Refectivity: Greater Than or Equal to Retinal Pigment Epithelium
OCT Presence of Retinal Pigment Epithelium Elevation (Drusen/PED): At Least One Definitive Core
OCT Presence of Retinal Pigment Epithelium Elevation (Drusen/PED): Focal High Reflectivity over Retinal Pigment Epithelium Elevation or Other Lesion
OCT Presence of Retinal Pigment Epithelium Elevation (Drusen/PED): Photoreceptor Thinning Above Retinal Pigment Epithelium Elevation
OCT Presence of Retinal Layer Atrophy or Absence: Foveal
OCT Presence of Retinal Layer Atrophy or Absence: Less Than or Equal to the Photoreceptor Layer
OCT Presence of Retinal Layer Atrophy or Absence: Photoreceptor Outer Segment Change/Loss
OCT Presence of Vitelliform Lesion
OCT Presence of Photoreceptor Thinning without Retinal Pigment Epithelium Elevation or Atrophy: Foveal
OCT Presence of Photoreceptor Thinning without Retinal Pigment Epithelium Elevation or Atrophy: Photoreceptor Outer Segment Changes/Loss
OCT Substructures Hyper-Reflective Foci Presence
OCT Substructures Hyper-Reflective Foci Below External Limiting Membrane Count up to 7 (n)
OCT Substructures Hyper-Reflective Foci Above External Limiting Membrane Count up to 7 (n)
OCT Substructures Hyper-Reflective Foci: Weighted Axial Distribution (AxD) Score (n+2m)
OCT Substructures Hyper-Reflective Foci: Mean Weighted Axial Distribution (mAxD) by External Limiting Membrane (AxD/(n+m))
OCT Substructures Hyper-Reflective Foci: Subretinal Drusenoid Deposits: 5 or more
OCT Substructures Choroidal Thickness in 3 mm
OCT Substructures Hyperreflective Foci: Choroidal Thickness 3 Subretinal Drusenoid Deposits Absent

© 2023 Dow ER et al. *JAMA Ophthalmology*.

eTable 3. A comparison of patient recruitment with or without the use of DeepGAze for a clinical trial targeting the progression of intermediate age-related macular degeneration (iAMD) to geographic atrophy (GA). The simulation assumes that 1000 patients would be recruited for the trial. Four different rates of per-year progression from iAMD to GA are provided that span the range of previously published estimates of baseline disease progression. Two operating points for the algorithm are demonstrated: one selected by Youden's index as the optimal operating point based on a balance of sensitivity and specificity; and a second operating point selected for high-specificity at an acceptable sensitivity for optimal clinical trial screening.

	Baseline incidence of progression from iAMD to GA (per year)	iAMD patients progressing to GA without AI enrichment (per year per 1000 patients)	Patients flagged as high-risk converters by DeepGAze (per 1000 patients)	Patients screened to reach trial enrollment of 1000 flagged as high-risk converters by DeepGAze	iAMD patients progressing to GA with DeepGAze enrichment (per year per 1000 patients)	Fold enrichment in patients with iAMD-to-GA progression with DeepGAze
**Youden operating point (DS2)**	0.75%	7.5	205	4,869	33	4.5
	1.50%	15	211	4,745	65	4.4
	3.00%	30	222	4,514	124	4.1
	5.00%	50	236	4,240	194	3.9
**Clinical-trial operating point (DS2)**	0.75%	7.5	28	35,395	157	20.9
	1.50%	15	33	30,764	273	18.2
	3.00%	30	41	24,384	432	14.4
	5.00%	50	52	19,102	564	11.3
**Clinical-trial operating point (DS3)**	0.75%	7.5	49	20,431	87	11.7
	1.50%	15	53	18,907	162	10.8
	3.00%	30	61	16,453	282	9.4
	5.00%	50	71	14,025	400	8.0

Youden operating point (DS2)	
**Sensitivity**	0.917
**Specificity**	0.800

Clinical-trial operating point (DS2)	
**Sensitivity**	0.591
**Specificity**	0.976

Clinical-trial operating point (DS3)	
**Sensitivity**	0.571
**Specificity**	0.955

© 2023 Dow ER et al. *JAMA Ophthalmology*.

eFigures

eFigure 1. a) Model architecture of the proposed multi-scan position-aware model for geographic atrophy (GA) prediction, simultaneously for diagnosis (current year) and prognosis (following year). The detail in the right-hand side shows the building blocks of the position-aware module used for view pooling. b) Learning with proactive pseudo-interventions (PPI). Saliency maps are obtained via Weight Backpropagation (WBP). Negative contrasts are constituted by masking out the saliency map from the input scans. Positive contrasts are constituted by either using the input scans without masking or by using a randomly selected mask. The multi-scan position-aware model performs predictions on both negative and positive contrasts.

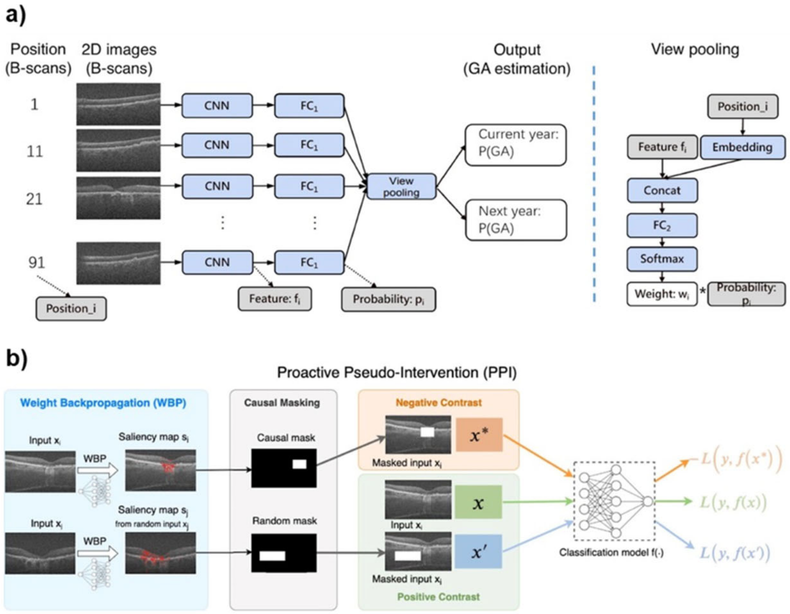

© 2023 Dow ER et al. *JAMA Ophthalmology*.

eFigure 2. Attention maps of eyes with current intermediate age-related macular degeneration (iAMD; A), that will progress to GA in one year (B), or that will remain iAMD the following year (C). The top image overlays DeepGAze attention mapping (red dots) over the unaltered bottom image. GA probability of the SD-OCT line scan and corresponding SD-OCT volume are denoted as p and p', respectively.

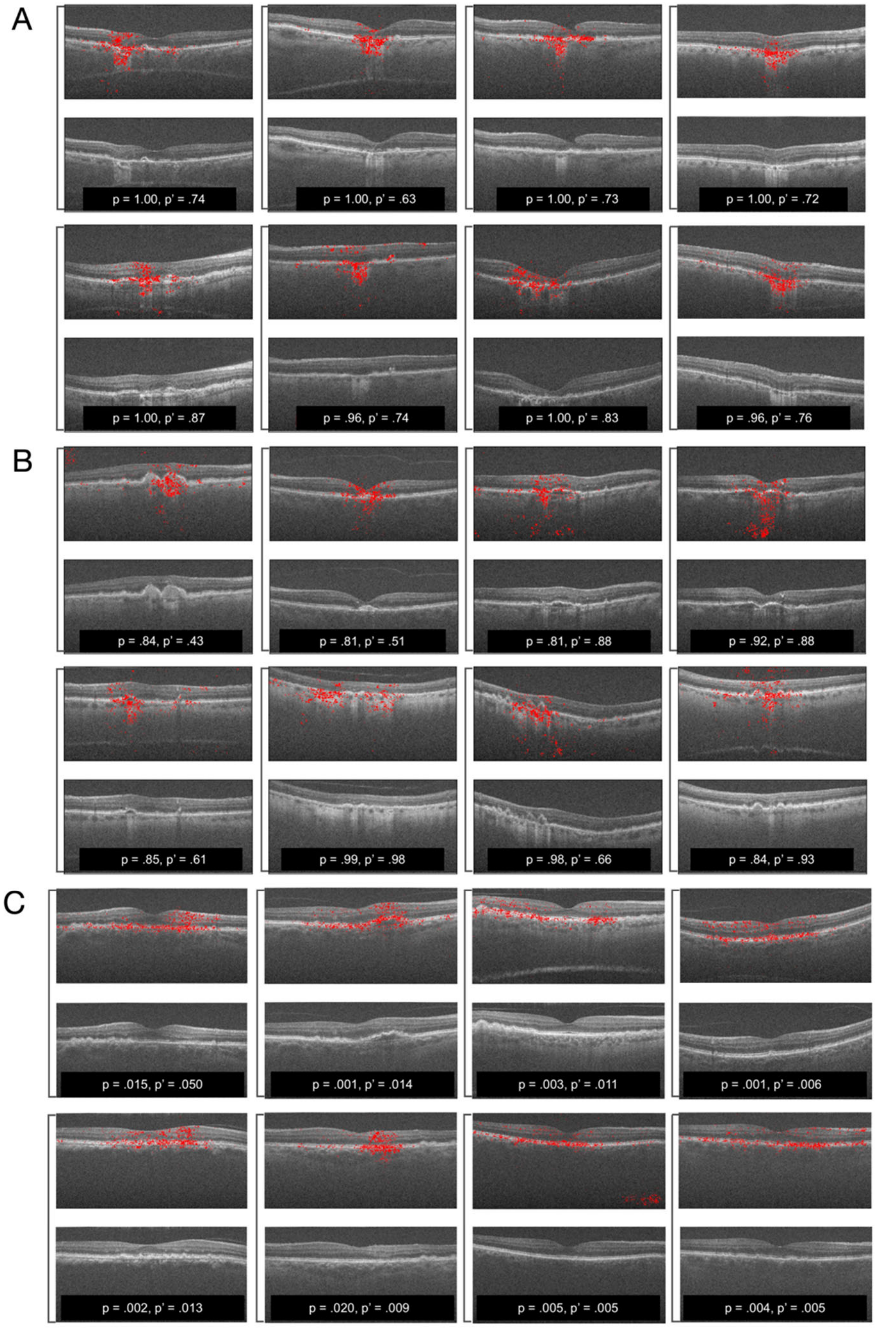

© 2023 Dow ER et al. *JAMA Ophthalmology*.

eFigure 3. Case study of individual eyes across scan volumes. (A) Case study of an eye with geographic atrophy (GA). Images in rows 2 and 4 are optical coherence tomography (OCT) scans of the same eye with GA. Images in rows 1 and 3 are their corresponding attention maps. GA probability of the scan is denoted as p. A histogram of p values for all 100 scans can be found in the bottom of the figure. (B) Case study of eye with intermediate age-related macular degeneration (iAMD) that will progress to GA in one year. Images in rows 2 and 4 are various SD-OCT scans from the same volume. Images in rows 1 and 3 are their corresponding attention maps. The probability of GA development in one year scan is denoted as p. The histogram of p values for all 100 scans can be found in the bottom of the figure.

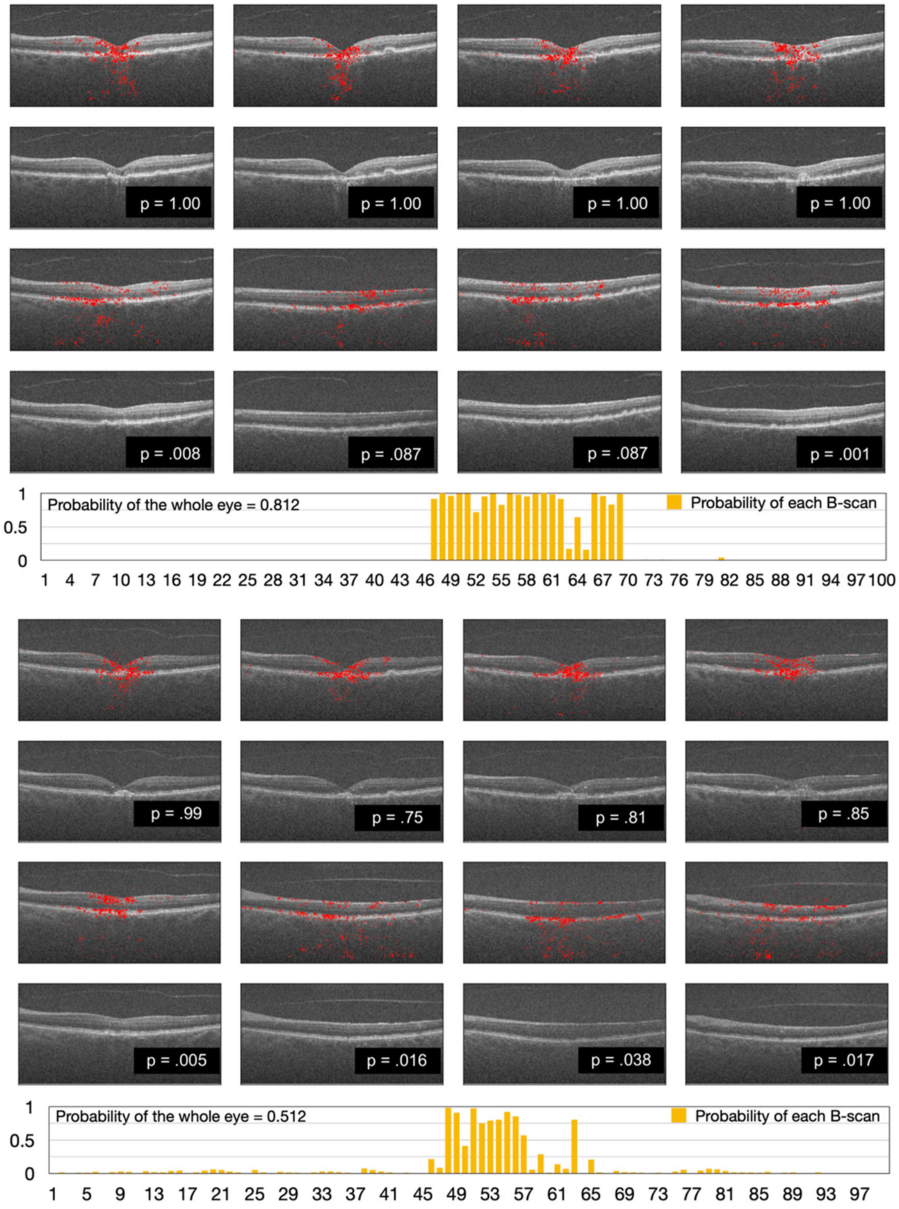

© 2023 Dow ER et al. *JAMA Ophthalmology.*

eFigure 4. Weight backpropagation attention maps generated from model inference on the external validation data set. The first and second rows are from eyes with intermediate age-related macular degeneration (iAMD) that will not progress to geographic atrophy (GA) within the next thirteen months; the third and fourth rows are from eyes that will progress to GA within the 1-13 months.

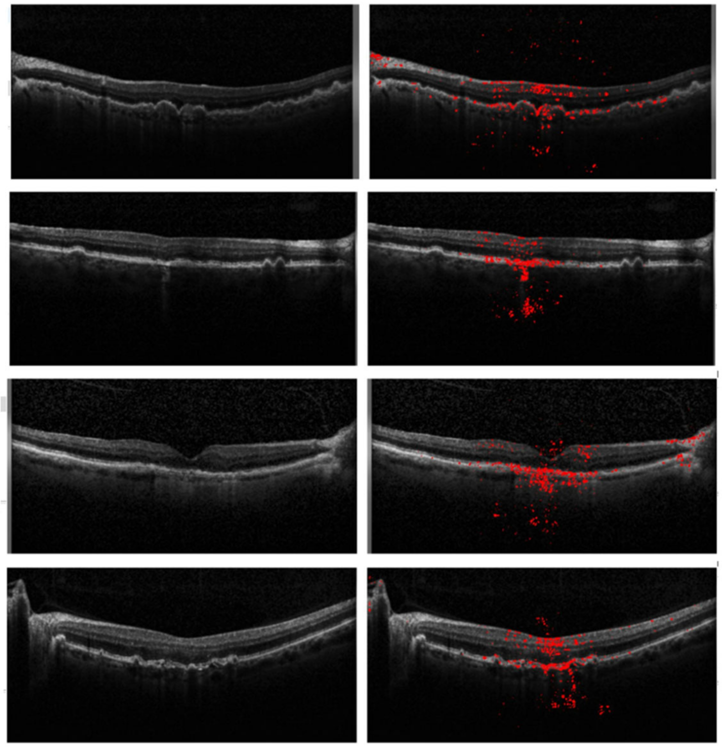

© 2023 Dow ER et al. *JAMA Ophthalmology.*

eFigure 5. Example predictions from DeepGAze. A-D. True positive prediction of progression to geographic atrophy (GA). Attention map (AB) shows clustering around the pigment epithelial defect (PED, blue) with overlying hyperreflective foci (HRF, purple) that collapses into a GA lesion (overlying magenta arrowhead) less than one year later. The lesion continues to expand during the subsequent three years. E-H. True negative prediction of nonprogression to GA shows attention directed to several PEDs as well as diffusely distributed along the outer retina. At 26 months after prediction, the eye remains without GA. I-L. False positive prediction. Model attention is diffusely distributed rather than concentrating over disease lesions. One year after prediction, the eye has not progressed, but just over two years later, the PED collapses resulting in a small GA lesion. M-P. False negative prediction. Model attention is anomalously clustered at an area of peripapillary atrophy rather than at the PED with underlying choroidal hypertransmission (violet) that will collapse into a GA lesion one year later. Subretinal drusenoid deposits are highlighted in green.

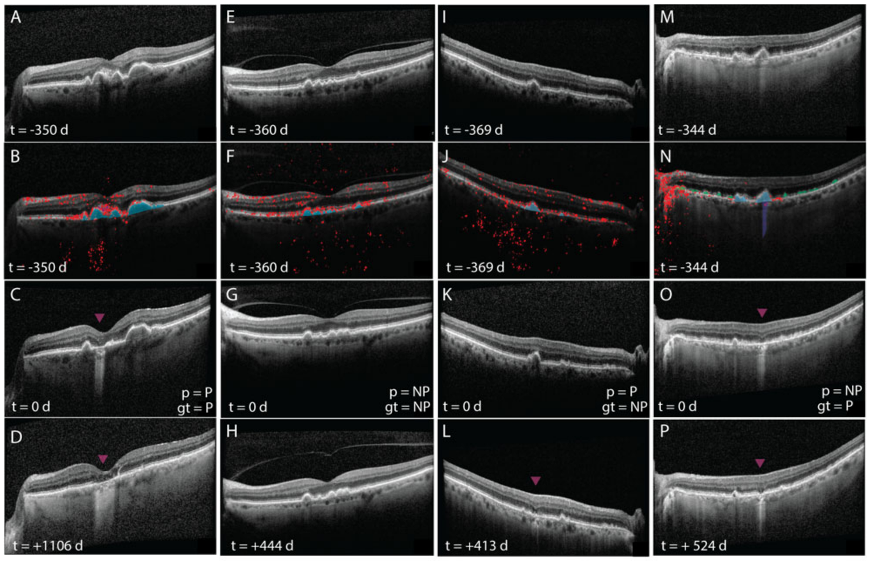

© 2023 Dow ER et al. *JAMA Ophthalmology.*

eFigure 6. Predicted pre-classification $p(GA)$ values (y-axis) grouped by scans (x-axis) and geographic atrophy (GA) status; orange designates the GA group and blue designates the control group. Lines and shaded areas represent mean and standard deviations, respectively, for cross-validated predictions obtained from the proposed model

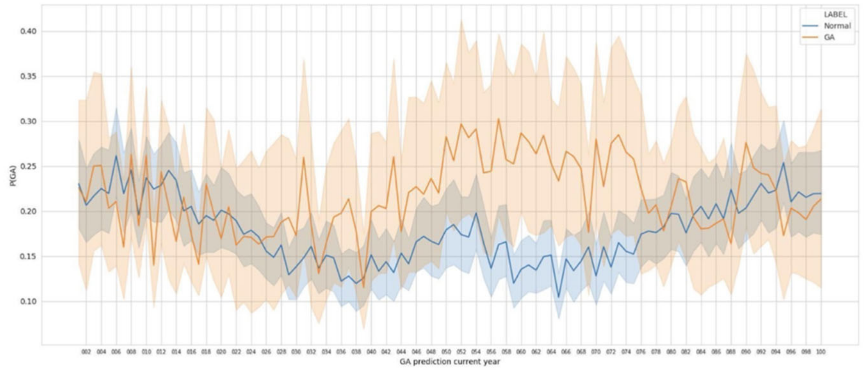

.

© 2023 Dow ER et al. *JAMA Ophthalmology*.

eMethods

AREDS-A2A Dataset

Over 7.4 years 316 patients with (1) bilateral large drusen > 125 μm or non-foveal GA, or (2) large drusen or non-foveal GA in one eye and advanced AMD (neovascularization or foveal GA) in the other eye were followed for conversion to foveal GA. Neovascularization or foveal GA at the outset were excluded as study eyes.[1,2] During the study period, 58 eyes progressed from iAMD to GA and 258 eyes remained as iAMD. In concordance with standard definitions,[3–7] OCT-GA was defined as the presence of the following three criteria: (1) RPE atrophy or absence, (2) choroid enhancement, and (3) outer plexiform layer (OPL) dipping towards the RPE over an area at least 175 μm.[8–10] SD-OCT volumes were graded by certified, blinded expert graders in the Duke Advanced Research in SS/SD-OCT Imaging Laboratory using the Duke Optical Coherence Tomography Retinal Analysis Program.[11–13]

Model

Geographic atrophy has been shown to begin in a perifoveal location, suggesting that SD-OCT scans from perifoveal regions will have preferential ability to identify GA.[8] To account for the varying predictability of each scan or "view" of the retina, we leveraged the multi-view CNN architecture,[7] which combined information from multiple "views" or scans of the retina to perform SD-OCT volume-level predictions. We proposed an end-to-end multi-scan position-aware volumetric image classification model. The model is presented in **eFigure 1.** Using the M=100 scans of an SD-OCT volume, for the i-th scan, the model used a shared CNN image feature extractor to obtain scan features (f_i) that were then fed to a fully connected (FC) layer[14,15] with sigmoid activation function[16] to obtain scan-wise pre-classification GA probabilities (p_i). Pre-classification probability (p_i) was defined as the initial probability of GA or progression to GA for the i-th scan prior to pooling views in subsequent steps. The CNN image feature extractor has the structure of the Inception V3 neural network, which was initialized with parameters from the Inception V3 architecture[17] pre-trained on natural images from ImageNet.[18] To visually interpret model predictions, the model generates attention maps via WBP,[19] which probabilistically masks out regions of the scan that do not contribute to the ability of the model to predict GA and the estimation of p_i. Subsequently, pre-classification GA probabilities (p_i) from different scans were aggregated, for which we considered different approaches, for instance, a simple average (i.e., mean pooling).

As shown in **eFigure 6**, some image scans were more informative of GA than others after model training (in terms of their p_i values) when examining the differences in distribution of the GA group relative to the controls, confirming that scan position may be leveraged for improved GA identification. Thus, we proposed the position-aware view pooling illustrated in the right panel of **eFigure 1a**, in which each scan is assigned a position identifier ranging from 1 to M. The model first used a transformation layer to embed the position identifier into a 6-dimensional positional feature vector e_i. Then, the feature vector f_i and positional feature e_i were concatenated and fed into a fully-connected layer to obtain $a_i = FC_2([f_i, e_i])$, which were progressed to attention weights w_i by feeding the a_i into a softmax function, so $\sum_{i=1}^{M} w_i = 1$. The final probability of GA for a given SD-OCT volume was the weighted summation of the attention weights w_i and corresponding pre-classification probabilities p_i for all scans, $p(GA) = \sum_{i=1}^{M} w_i p_i$. It is worth noting that the positional features and attention weights allow the model to accommodate volumes of different sizes, thus not necessarily restricted to M=100.

The model was trained in a contrastive learning manner as shown in **eFigure 1** to maximize the weighted binary cross-entropy loss, (i.e., $L(y, f(x))$, the likelihood that scans from SD-OCT inputs, x, were correctly assigned (prognosticated) to either the GA or control groups, y, in the assessment of the following year, while encouraging that *i*) regions masked-out by the saliency maps, x^*, which we call *negative contrasts*, were not informative of GA, and *ii*) unmasked input scans, x, and regions masked-out by a randomly assigned saliency map, x', which we call *positive contrasts*, did not affect the model's ability to predict GA. This strategy, termed Proactive Pseudo Intervention (PPI) learning, was previously described.[19] Note that positive and negative contrasts consist of mutually exclusive image regions and that negative contrasts are created to prevent that learned saliency maps include non-predictive regions or image regions spuriously associated with GA. Additionally, the model predicted the probability of GA in the current year. We allowed the model to learn $p(GA)$ in the current and following year simultaneously to encourage a more informative and robust feature extractor.

The small sample-size of the dataset (316 iAMD patients, 1085 volumetric SD-OCT scans) placed limitations on the model to be trained. One way to improve model performance is by leveraging additional data to perform multi-task learning.[18] We employed a publicly available SD-OCT dataset[20] consisting of 108,312 individual SD-OCT B-scans (not full SD-OCT volumes) from 4,686 individuals. These images were labeled for the following

classification endpoints: choroidal neovascularization (CNV), diabetic macular edema (DME), drusen, and control. The single-scan B-scan images were higher resolution than those from the 100-scan SD-OCT volumes acquired in the A2A SD-OCT Study. Implementing multi-task learning, shared information from these related tasks (predicting iAMD progression to GA and identification of various pathologic features) can be leveraged to improve the model performance on our GA prediction task.[20] This multi-task approach shared the CNN image feature extractor (Inception V3) but used separate FC layers for these three tasks: (1) current GA prediction (2) progression to GA in the following year, and (3) CNV, DME, drusen and control prediction.

Due to the infrequency of progression from iAMD to GA, several approaches were tested to bolster model performance on the small data set with an ablation design to determine the contribution of each approach. First, CNN classification on single B-scans was compared to classification across all B-scans in the SD-OCT volume ("multi-scan"). All versions of the multi-scan approach outperformed all versions of the single-scan approach including the single-scan models trained on a large public database of individual SD-OCT B-scans (**eTable 1**). Second, implementation of an embedding that made the model position-aware, rather than considering the B-scans as an unordered set, also boosted performance (**eTable 1**). Next, for each of the 100 B-scans in an SD-OCT volume pre-classification probabilities of GA were generated by the model and then plotted. We found that the average probability of GA was substantially higher in the GA group than that of the iAMD group, and moreover, that scans in the center (lines 35-75) were much more informative of GA presence compared to those at the extremes (1-34 and 76-100) (**eFigure 1**). Therefore, probabilities from scans 35-75 were given greater weight in the final classification than peripheral scans. Finally, training the model using PPI, a form of contrastive learning, further improved classification performance compared to models not trained with this technique (**Figure 1, eTable 1**). Comparison between the final model and the next closest by AUROC resulted in $p < .001$ using the Delong test for receiving operating characteristic comparisons.[21]

The model was coded in PyTorch and trained with the Adam Optimizer[22] on a GPU TITAN Xp for 100 epochs with a learning rate of 0.0005 for pre-trained feature exactor (0.005 for fully connected layers) and a decay of 0.5 applied to the learning rate at every 10 epochs. The dataset available for model training consisted of 108,500 512x1000 pixels SD-OCT scans corresponding to 1085 individuals, 28% of which (30,400 scans) correspond to GA patients. A major concern associated with limited labeled data was over-fitting, meaning the model performed very well on the training data (over-fitting the observed data), but performed poorly on testing (unobserved or new data). Consequently, we estimated model performance via 5-fold cross-validation to maximize the data available for model training while still being able to properly estimate performance characteristics. Specifically, we reported AUROC,[23] as well as Standard Deviation (SD) over 5-fold to represent performance variability. The statistical significance of the difference between ROC curves for different models was quantified with the DeLong test.[24] Further, we presented confusion matrices and their summaries (sensitivity, specificity, PPV, NPV, and accuracy) obtained by thresholding the predictions, values, from the model with thresholds estimated via Youden's index.[25] All performance metrics were computed for both tasks: (1) current presence of GA and (2) progression to GA in one year.

In order to justify the design choices in the proposed multi-scan position-aware model trained with PPI, we also considered simplified versions of this model (i.e., an ablation study): without PPI learning, only using the center of the SD-OCT volume (scans 35-75) without the position-aware pooling, training with single scans (in contrast with "multi-scan" or multiple views) and averaging the predictions, using a feature encoder pretrained on a separate set SD-OCT scans, and using a feature encoder pretrained on ImageNet. Technical details of the model have been previously reported.[19] Additionally, human-annotated features were fed into the pre-prediction layer of the model to determine if it could improve model accuracy. These human-annotated features have been previously published.[1,9,26–29] For model validation, SD-OCT volume scans from the independent validation data sets, Data Set 2 and 3, described above were input to the final model (multi-scan, position-aware model trained with PPI) after CLAHE image normalization.

Supplemental References

1. Folgar FA, Chow JH, Farsiu S, et al. Spatial correlation between hyperpigmentary changes on color fundus photography and hyperreflective foci on SDOCT in intermediate AMD. *Invest Ophthalmol Vis Sci*. 2012;53(8):4626-4633. doi:10.1167/IOVS.12-9813
2. Ferris FL, Davis MD, Clemons TE, et al. A simplified severity scale for age-related macular degeneration: AREDS Report No. 18. *Arch Ophthalmol*. 2005;123(11):1570-1574. doi:10.1001/ARCHOPHT.123.11.1570
3. Wu Z, Luu CD, Ayton LN, et al. Optical coherence tomography-defined changes preceding the development of drusen-associated atrophy in age-related macular degeneration. *Ophthalmology*. 2014;121(12):2415-2422. doi:10.1016/J.OPHTHA.2014.06.034
4. Sadda SR, Chakravarthy U, Birch DG, Staurenghi G, Henry EC, Brittain C. CLINICAL ENDPOINTS FOR THE STUDY OF GEOGRAPHIC ATROPHY SECONDARY TO AGE-RELATED MACULAR DEGENERATION. *Retina*. 2016;36(10):1806-1822. doi:10.1097/IAE.0000000000001283
5. Holz FG, Sadda SVR, Staurenghi G, et al. Imaging Protocols in Clinical Studies in Advanced Age-Related Macular Degeneration: Recommendations from Classification of Atrophy Consensus Meetings. *Ophthalmology*. 2017;124(4):464-478. doi:10.1016/J.OPHTHA.2016.12.002
6. Fleckenstein M, Schmitz-Valckenberg S, Adrion C, et al. Tracking progression with spectral-domain optical coherence tomography in geographic atrophy caused by age-related macular degeneration. *Invest Ophthalmol Vis Sci*. 2010;51(8):3846-3852. doi:10.1167/IOVS.09-4533
7. Fleckenstein M, Schmitz-Valckenberg S, Martens C, et al. Fundus autofluorescence and spectral-domain optical coherence tomography characteristics in a rapidly progressing form of geographic atrophy. *Invest Ophthalmol Vis Sci*. 2011;52(6):3761-3766. doi:10.1167/IOVS.10-7021
8. Leuschen JN, Schuman SG, Winter KP, et al. Spectral-domain optical coherence tomography characteristics of intermediate age-related macular degeneration. *Ophthalmology*. 2013;120(1):140-150. doi:10.1016/J.OPHTHA.2012.07.004
9. Veerappan M, El-Hage-Sleiman AKM, Tai V, et al. Optical Coherence Tomography Reflective Drusen Substructures Predict Progression to Geographic Atrophy in Age-related Macular Degeneration. *Ophthalmology*. 2016;123(12):2554-2570. doi:10.1016/J.OPHTHA.2016.08.047
10. Sleiman K, Veerappan M, Winter KP, et al. Optical Coherence Tomography Predictors of Risk for Progression to Non-Neovascular Atrophic Age-Related Macular Degeneration. *Ophthalmology*. 2017;124(12):1764-1777. doi:10.1016/J.OPHTHA.2017.06.032
11. Holz FG, Strauss EC, Schmitz-Valckenberg S, Van Lookeren Campagne M. Geographic Atrophy: Clinical Features and Potential Therapeutic Approaches. *Ophthalmology*. 2014;121(5):1079-1091. doi:10.1016/J.OPHTHA.2013.11.023
12. Lindblad AS, Lloyd PC, Clemons TE, et al. Change in area of geographic atrophy in the Age-Related Eye Disease Study: AREDS report number 26. *Arch Ophthalmol*. 2009;127(9):1168-1174. doi:10.1001/ARCHOPHTHALMOL.2009.198
13. Wong WL, Su X, Li X, et al. Global prevalence of age-related macular degeneration and disease burden projection for 2020 and 2040: a systematic review and meta-analysis. *Lancet Glob Health*. 2014;2(2). doi:10.1016/S2214-109X(13)70145-1
14. Basaia S, Agosta F, Wagner L, et al. Automated classification of Alzheimer's disease and mild cognitive impairment using a single MRI and deep neural networks. *Neuroimage Clin*. 2019;21. doi:10.1016/J.NICL.2018.101645
15. Liu M, Li F, Yan H, et al. A multi-model deep convolutional neural network for automatic hippocampus segmentation and classification in Alzheimer's disease. *Neuroimage*. 2020;208. doi:10.1016/J.NEUROIMAGE.2019.116459
16. Hastie T, Tibshirani R, Friedman J. *The Elements of Statistical Learning: Data Mining, Inference, and Prediction, Second Edition*.; 2016.
17. Szegedy C, Vanhoucke V, Ioffe S, Shlens J, Wojna Z. Rethinking the Inception Architecture for Computer Vision. *Proceedings of the IEEE Computer Society Conference on Computer Vision and Pattern Recognition*. 2015;2016-December:2818-2826. doi:10.1109/CVPR.2016.308
18. Sun C, Shrivastava A, Singh S, Gupta A. Revisiting Unreasonable Effectiveness of Data in Deep Learning Era. *Proceedings of the IEEE International Conference on Computer Vision*. 2017;2017-October:843-852. doi:10.1109/ICCV.2017.97
19. Wang D, Yang Y, Tao C, et al. Proactive Pseudo-Intervention: Causally Informed Contrastive Learning For Interpretable Vision Models. Published online December 6, 2020. Accessed June 3, 2023. https://arxiv.org/abs/2012.03369v2

© 2023 Dow ER et al. *JAMA Ophthalmology.*

20. Kermany DS, Goldbaum M, Cai W, et al. Identifying Medical Diagnoses and Treatable Diseases by Image-Based Deep Learning. *Cell*. 2018;172(5):1122-1131.e9. doi:10.1016/J.CELL.2018.02.010
21. DeLong ER, DeLong DM, Clarke-Pearson DL. Comparing the areas under two or more correlated receiver operating characteristic curves: a nonparametric approach. *Biometrics*. 1988;44(3):837. doi:10.2307/2531595
22. Kingma DP, Ba JL. Adam: A method for stochastic optimization. *3rd International Conference on Learning Representations, ICLR 2015 - Conference Track Proceedings*. Published online 2015:1-15.
23. Fawcett T. An introduction to ROC analysis. *Pattern Recognit Lett*. 2006;27(8):861-874. doi:10.1016/j.patrec.2005.10.010
24. Demler O V., Pencina MJ, D'Agostino RB. Misuse of DeLong test to compare AUCs for nested models. *Stat Med*. 2012;31(23):2577-2587. doi:10.1002/SIM.5328
25. Youden WJ. Index for rating diagnostic tests. *Cancer*. 1950;3(1):32-35. doi:10.1002/1097-0142(1950)3:1<32::AID-CNCR2820030106>3.0.CO;2-3
26. Lad EM, Sleiman K, Banks DL, et al. Machine learning OCT predictors of progression from intermediate age-related macular degeneration to geographic atrophy and vision loss. *Ophthalmology science*. 2022;2(2):100160. doi:10.1016/J.XOPS.2022.100160
27. Christenbury JG, Folgar FA, O'Connell R V., Chiu SJ, Farsiu S, Toth CA. Progression of intermediate age-related macular degeneration with proliferation and inner retinal migration of hyperreflective foci. *Ophthalmology*. 2013;120(5):1038-1045. doi:10.1016/J.OPHTHA.2012.10.018
28. Farsiu S, Chiu SJ, O'Connell R V., et al. Quantitative classification of eyes with and without intermediate age-related macular degeneration using optical coherence tomography. *Ophthalmology*. 2014;121(1):162-172. doi:10.1016/J.OPHTHA.2013.07.013
29. Folgar FA, Yuan EL, Sevilla MB, et al. Drusen Volume and Retinal Pigment Epithelium Abnormal Thinning Volume Predict 2-Year Progression of Age-Related Macular Degeneration. *Ophthalmology*. 2016;123(1):39-50.e1. doi:10.1016/J.OPHTHA.2015.09.016

© 2023 Dow ER et al. *JAMA Ophthalmology.*
