# Supplementary material for: A Deep-Learning Algorithm to Predict Short-Term Progression to Geographic Atrophy on Spectral-Domain Optical Coherence Tomography
Source: JAMA Ophthalmol. 2023 Oct 19;141(11):1052–61. doi: 10.1001/jamaophthalmol.2023.4659 (PMC10587827; doi:10.1001/jamaophthalmol.2023.4659)
Supplement: Supplement 2. — Data sharing statement [file jamaophthalmol-e234659-s002.pdf]

Data Sharing Statement

Dow. A Deep-Learning Algorithm to Predict Short-Term Progression to Geographic Atrophy on Spectral-Domain Optical Coherence Tomography. *JAMA Ophthalmol*. Published October 19, 2023. doi:10.1001/jamaophthalmol.2023.4659

Data
Data available: No

Additional Information
Explanation for why data not available: Non-HPI data can be shared upon request.
